# The role of Juniperus Macrocarpa extract as anti-inflammatory and antioxidant on methotrexate-induced acute liver injury in rat model

**DOI:** 10.12688/f1000research.158839.1

**Published:** 2025-01-27

**Authors:** Shahad Hassan Hadi, Mohammed Qasim Yahya Malallah A. Al-Atrakji

**Affiliations:** 1 Msc candidate (Pharmacology), Department of Pharmacology, College of Medicine, University of Baghdad, Baghdad, Baghdad Governorate, Iraq; 2 PhD (Pharmacology), Department of Pharmacology, College of Medicine, University of Baghdad, Baghdad, Baghdad Governorate, Iraq

**Keywords:** Juniperus Macrocarpa; Methotrexate; hepatotoxicity; Oxidative stress; Apoptosis; Cytokines

## Abstract

**Background:**

Methotrexate (MTX) is an antifolate medication indicated to treat an array of tumors and autoinflammatory maladies. MTX may exhibit harmful impacts on multiple organs, especially liver injury and cirrhosis. Juniperus macrocarpa is a medicinal herb enriched with polyphenols and flavonoids featuring robust anti-inflammatory and antioxidative benefits.

**Objective:**

To evaluate the hepatoprotective effects of Juniperus macrocarpa aqueous extract on MTX-aggravated liver toxicity.

**Methods:**

The study involved 20 male middle-aged albino rats, arbitrarily allocated into 4 groups of 5 animals each. Group 1 (control) were given distilled water (DW) once daily for two weeks. Group 2 (MTX) got an intraperitoneal single dose of MTX (20 mg/kg) for two weeks. Rats in groups 3 and 4 were given daily dosages of 100 mg and 200 mg of Juniperus macrocarpa aqueous extract, respectively, for two weeks before receiving a single intraperitoneal MTX injection.

**Results:**

Juniperus macrocarpa extracts at both low and high doses substantially alleviated the MTX-provoked biochemical alterations, as evidenced by decreased levels of inflammatory parameters including TNF-α and IL-6 and hepatic enzymes including ALT, AST, and ALP. Juniperus macrocarpa also significantly boosted levels of the anti-oxidant enzymes like SOD and GPX. Moreover, Juniperus macrocarpa extract attenuated congestive and degenerative hepatic changes, as indicated by improved histopathological findings.

**Conclusion:**

The anti-oxidative and anti-inflammatory activities of Juniperus macrocarpa extract are a promising approach for ameliorating MTX-aggravated hepatotoxicity.

## Introduction

Drug-induced liver Injury (DILI) is a generic term encompassing the spectrum of pathological responses that occur in the liver as a result of exposure to chemical compounds that have the potential to be destructive to the liver. ^1– 3^ DILI is a serious risk that is a subject of great concern, and it is a risk that is present in both the preclinical drug development process and as a primary source of candidate drug attrition. ^2, 4– 6^ One of the most common reasons for acute liver failure (ALF) in developed countries is thought to be pharmaceutical intoxication, which is then followed by acute hepatitis B. ^7, 8^

Methotrexate (MTX) is a potent anticancer drug that acts as an antagonist to folic acid. It has proven to be highly effective in treating various types of cancer, including lymphoma, leukemia, breast cancer, and osteosarcoma. ^9– 11^ Moreover, MTX is extensively utilized in the management of autoimmune conditions such as rheumatoid arthritis and psoriasis. ^12– 15^ Despite its clinical efficacy, the use of MTX has been associated with hepatotoxicity, which is a major adverse effect that manifests as tissue destruction, liver dysfunction, fibrosis, and cirrhosis, along with notable modifications in the function of the liver, biochemical indicators, and histological structure. ^16– 18^ The exact mechanism by which MTX-induced liver damage is not understood; however, several researchers attributed MTX-induced hepatotoxicity to oxidative stress, apoptosis, and inflammation. ^19– 21^

Several disorders have been successfully treated with herbal treatment, which involves the use of items derived from plants. When compared to synthetic medication, these natural remedies are safer and have fewer adverse effects than the synthetic medicine. ^22– 32^ Therefore, several researchers tried to evaluate the effectiveness of herbal products such as Thymus Vulgaris, ^19^ Origanum onites essential oil, ^33^ Crocus sativus stigma, ^21^ Syzygium aromaticum essential oil extract, ^34^ and Moringa oleifera leaf extract ^35^ in MTX-induced hepatotoxicity.

Juniperus Macrocarpa is a. plant growing in Turkey and used for various medicinal purposes in traditional folk medicine. ^36– 38^ Phytochemical identification of Juniperus Macrocarpa extract revealed that it is rich in phenolic acids (such as protocatechuic acid and gallic acid), flavonoids (such as rutin, catechin, quercitrin, epicatechin, luteolin, and naringenin), and coumarins such as umbelliferone. The relative amount of active ingredient in the extract is dependent on the plant species and cultivation area. ^39– 41^

To the best of our knowledge, there have been no previous studies determined the potential effects of Juniperus Macrocarpa extract in the attenuation of MTX-exacerbated hepatotoxicity. The objective of this research was to assess whether or not pre-treatment with Juniperus Macrocarpa extract is effective in amelioration of MTX-induced hepatotoxicity via investigating the effects of the extract on the serum levels of the antioxidant enzymes, inflammatory markers, hepatic enzymes, and histological findings.

## Methods

### Experimental animals

Twenty male albino rats aged between 6 and 12 months with an average weight of 220 ± 30 g, were used in this study. The animals were acquired from the animal house of the Iraqi Center for Cancer Research and Medical Genetics– Baghdad. They were placed in polyethylene cages with stainless steel covers and kept for acclimatization for one week before the experiment. They were maintained in standard laboratory conditions (232°F, 12-hour light-dark cycle) and had free access to food from a chow pallet and tap water. The study was started from December 31, 2023, to July 1, 2024. This study was approved by the ethical committee for experimental studies at the College of Medicine/University of Baghdad.

### Drugs and reagents

Juniperus Macrocarpa plant was obtained from the Department of Pharmacognosy /College of Pharmacy/Baghdad University, Iraq. Methotrexate Methotrexate (MTX) was purchased from Medwise Healthcare Limited, 843 Finchley Road, London, UK. Catalog Number: M1435. Other substances were bought from well-known manufacturers.

## Methods

### Drug preparations

We used the dose of MTX required to induce hepatotoxicity in rats as 20 mg/kg (the quantity around 4.4 mg per rat), which was estimated according to previous studies. ^42^ The dose was calculated according to the rat’s body weight and given intraperitoneally.

Furthermore, an oral solution of Juniperus macrocarpa was prepared by estimating the quantity of Juniperus macrocarpa based on the findings of an oral acute toxicity study in which rats were given a dosage of 2000 mg/kg and exhibited no indication of toxicity in their bodies. Therefore, the study utilized one-tenth of this dose, which is equivalent to 200 mg/kg as a high dose, and half of the one-tenth as a low-level dose, to validate the safety of the substance, as stated by another study. ^43, 44^ An oral solution of the powder was made by combining powder Juniperus Macrocarpa with distilled water to provide a concentration of 40 mg/mL, for experimental rats with an average weight of 220 ± 30 g, a volume of 1 ml is necessary to provide a dose of 200 mg/ mL and 0.5 mL to provide a dose of 200 mg/mL according to the prescribed protocol.

### Experimental design

The preset study was performed at the Department of Pharmacology, College of Medicine, University of Baghdad, and the Iraqi Center for Cancer and Medical Genetic Research. The twenty rats were randomly divided into four groups with a total of five rats in each group. Participants who were a part of the experimental groups included the following individuals:

• **Group I (apparently healthy) group**(n=5): consisted of normal, healthy rats that were kept under normal laboratory conditions and received 1 mL of distilled water orally for 13 days by oral gavage.
• **Group II (Induction) group**(n=5): rats were adminstrated pre-treatment with 1 mL of distilled water orally for 13 days by oral gavage and 20mg/kg of Methotrexate intraperitoneally on day 13 of the study. ^42^
• **Group III (**low-dose Juniperus Macrocarpa extract-treated group)(n=5) rats were given Juniperus Macrocarpa extract (100 mg/kg) for 13 days by oral gavage and MTX intraperitoneally (as in Group II) on day 13 of the study.
• **Group IV** (high-dose Juniperus Macrocarpa extract-treated group)(n=5), rats were given Juniperus Macrocarpa extract (200 mg/kg) for 13 days by oral gavage and MTX intraperitoneally (as in Group II) on day 13 of the study.

### Anesthetic/Method of euthanasia

All precautions were taken to guarantee the wellbeing of the rats being studied and to limit any and reduce any pain, grief, or suffering. Attempts involved proper ventilation, typical cage disinfection, wood husk replacement every two days, attentive animal transportation. After 24 hours from the last MTX administration, the animals were euthanized using intraperitoneal anesthesia with 87 mg/kg of ketamine and 13 mg/kg of xylazine. ^45– 49^

### Tissue and blood sample Collection

The blood samples were obtained using direct cardiac puncture using and transferred into gel tubes. Subsequently, the tubes were centrifuged at a speed of 3000 revolutions per minute for a duration of 10 minutes. ^50– 53^ Following complete blood separation, the serum was extracted, placed into 2 mL non-treated plastic tubes, and stored at a temperature of -20 °C for subsequent analysis. ^54– 56^

Following the collection of the blood sample, the liver tissues were then isolated and rinsed with distilled water and preserved in a 10% formalin solution to improve and maintain the tissue structure while preventing degradation by lysosomal enzymes. This was done in preparation for a histopathological examination. ^57– 61^

The serum concentrations of superoxide dismutase (SOD) and glutathione peroxidase (GPx), as well as tumor necrosis factor-alpha (TNF-α) and interleukin-6 (IL-6), were measured for all experimental groups (1-4). The ELISA kits used in this study were commercially available and followed the instructions provided by the manufacturer (Cloud-Clone Crop ^®^ Laboratory, China). The initial phase in the experiment is to add anti-biomarker antibodies to a 96-well plate. Samples and standards were added to the wells, and the wrapping antibodies attracted any circulatory TNF-α, IL-β, SOD, or GPx to the wells. After extracting the unattached biotin-linked antibody, streptavidin and horseradish peroxidase (HRP) were carefully placed onto the plate. ^62– 65^ The number of several bio-markers in every specimen was calculated via comparison of optical density to conventional curves. The ceramic plates were washed again, and then TMB-substrate mixtures were applied to indicate the paired indicator amounts using the color produced. The absorbance of the samples was quantified using a microplate reader spectrophotometer. The color amplitude is computed at 450 nm as the color transitions from blue to yellow with a stopping solution. ^66– 69^

### Assessment of hepatic marker enzymes

The serum levels of aspartate aminotransferase (AST), alanine aminotransferase (ALT), and alkaline phosphatase (ALP) were quantified using the automated Flexor-EL80 system manufactured by Vitalab. 500μL of serum was added and incubated for 30 minutes before being read. ^3, 5, 70– 72^

### Assessment of histopathological changes

The liver for all experimental groups (1-4) were collected 24 hours after MTX injection on day 14 of the experiment. Following the administration of ketamine and xylazine anesthesia, the liver was extracted and preserved in 10% formalin for histological inspection. ^57, 73– 75^ The study utilized the conventional paraffin-embedded approach to prepare liver tissue for microscopic analysis. ^76– 78^ Afterward, the samples were immersed in paraffin, cut into slices that were 5 μm in thickness, and then treated with hematoxylin and eosin (H&E) stain. The histological slides were analyzed using standard light microscopy techniques at GENEX Laboratories in the USA. An experienced pathologist examined the slides without any prior knowledge, and only one sample slide was selected for each group. ^79– 82^ The semi-quantitative score compromises several categories including hepatocyte regeneration and necrosis, central and portal veins dilation and congestion, degree of fibrosis, infiltration of MNCs, and proliferation of cholangiocytes was evaluated by ranking the severity of hepatocyte necrosis, fibrosis (collagen deposition), cellular infiltration, apoptosis, and fatty alteration from liver sections were graded from 1 (minimum), 2 (mild), 3 (moderate), and 4 (marked). ^83– 86^ The total score for each slide was calculated and the median score with interquartile range was obtained.

### Statistical analysis

Graph Pad Prism 9 was used to conduct the statistical analysis. The median and interquartile ranges were utilized by the histolpathogical scoring system; however, the mean and standard deviation were calculated for all other data. The analysis of variance (ANOVA) and the post hoc Tukey’s tests were utilized to ascertain the group relationships. To achieve statistical significance, the P-value needed to be lower than 0.05. The non-parametric Kruskal-Wallis test was followed by Dunn’s multiple comparisons test to analyze the scores obtained from the histological groups. ^87^

## Results

**Figure 1.**
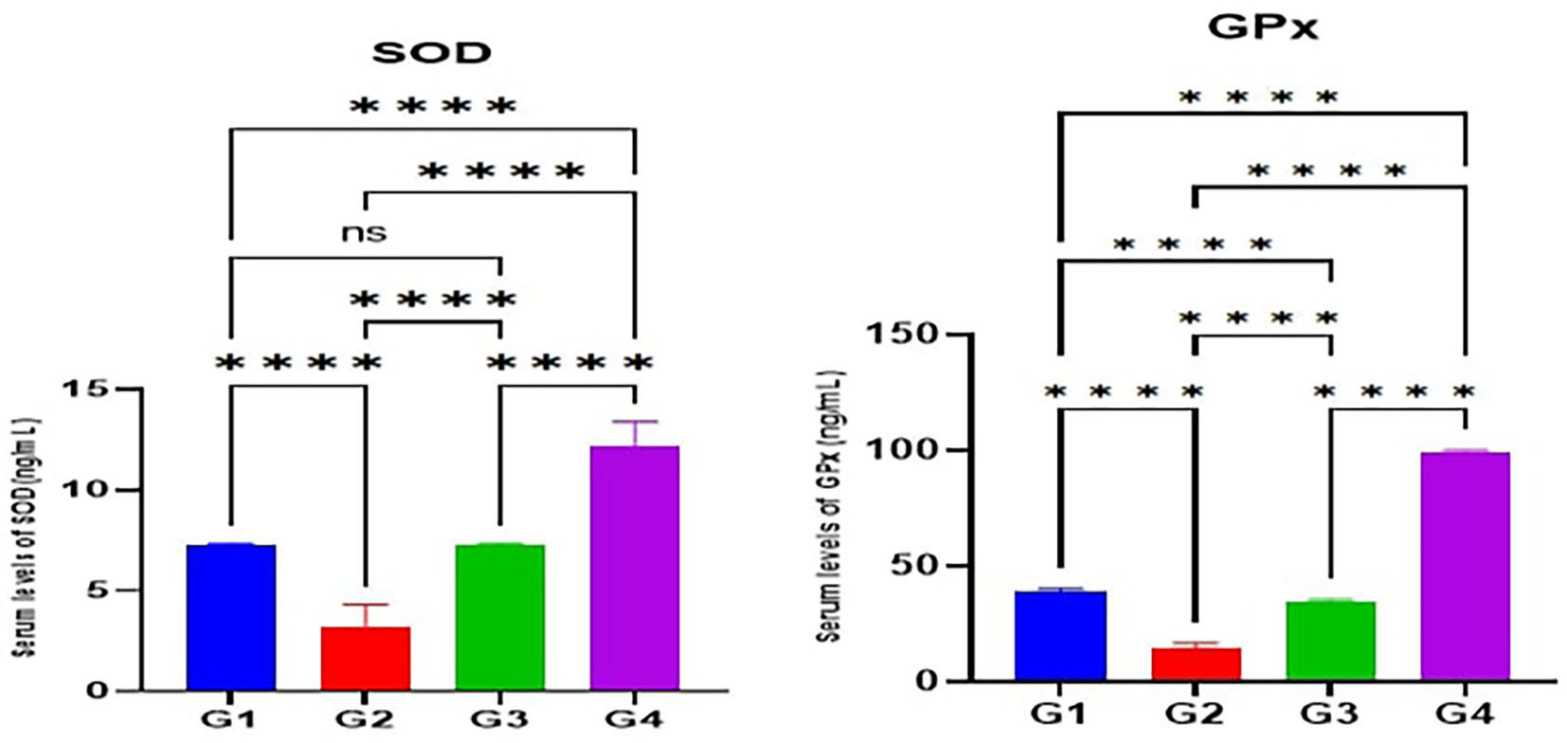
Effects of studied medications on serum levels of antioxidant enzymes SOD and GPx. Data are indicated as mean ±SD; ns (non-significant) = p > 0.05; ****= significant differences (p < 0.0001).

According to the results of the current study, the serum levels of antioxidant enzymes (SOD and GPx) in the MTX induction group (G2) were significantly lower (p<0.05) than the control(G1). Furthermore, the serum levels of SOD and GPx in Juniperus Macrocarpa extract-treated groups G3 and G4 were significantly higher (p<0.05) than the MTX induction group (G2). Also, the serum levels of both SOD and GPx in G4 were significantly higher (p<0.05) than in G3. Additionally, the serum levels of both SOD and GPx in G4 were significantly higher (p<0.05) than the control group (G1) as shown in Figure 1.

**Figure 2.**
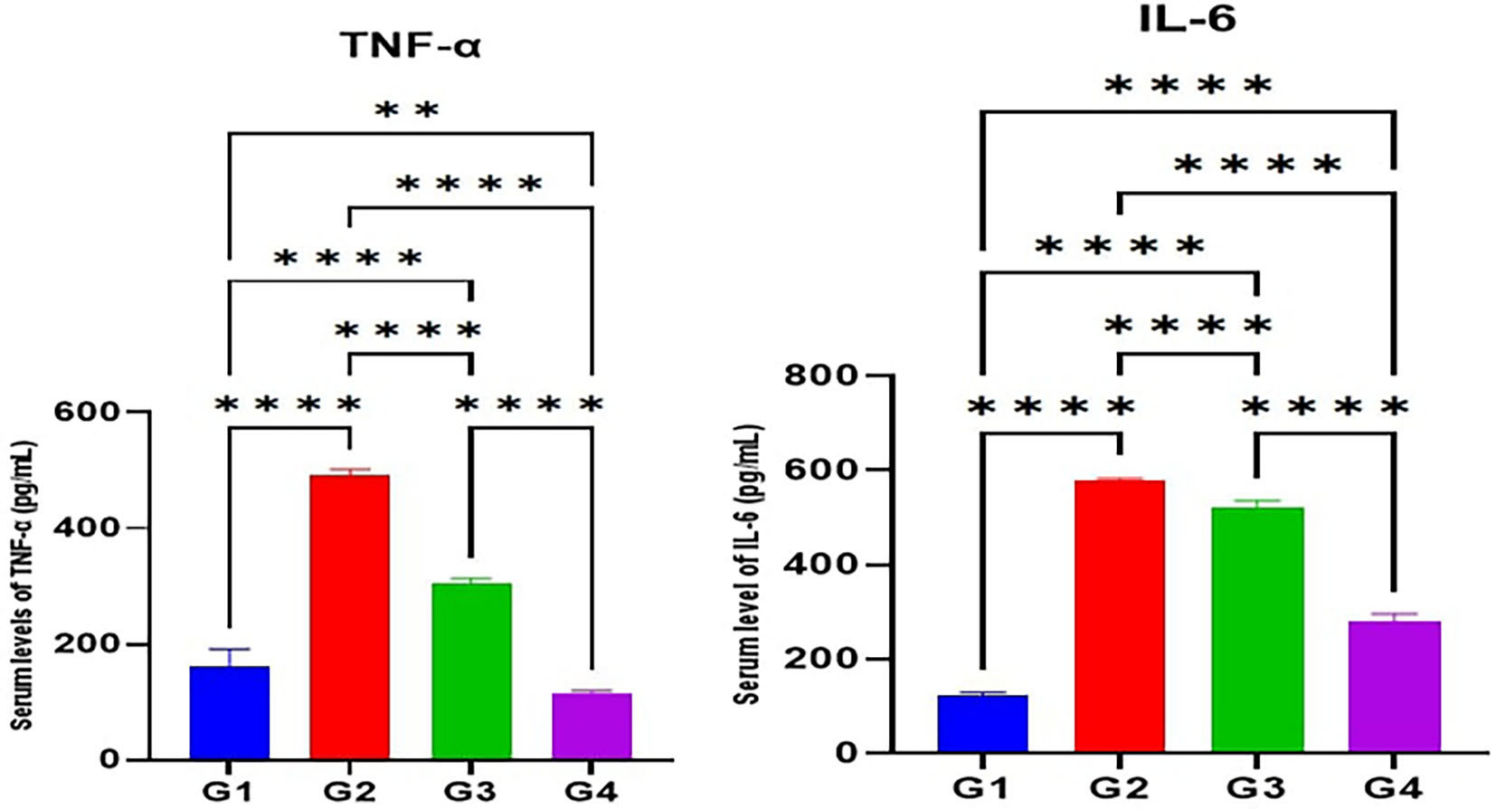
Effects of studied medications on serum levels of inflammatory cytokines TNF-α and IL-6. Data are indicated as mean ±SD; **= significant differences (p < 0.01); ****= significant differences (p < 0.0001).

According to the results of the current study, the serum levels of TNF-α and IL-6 in the MTX induction group (G2) were significantly higher (p<0.05) than the control (G1). Furthermore, the serum levels of TNF-α and IL-6 in Juniperus Macrocarpa extract-treated groups G3 and G4 were significantly lower (p<0.05) than the MTX induction group (G2). Also, the serum levels of both TNF-α and IL-6 in Juniperus Macrocarpa extract-treated groups G4 (200 mg/kg) were significantly lower (p<0.05) than in Macrocarpa extract-treated groups G3(100 mg/kg). However, the serum levels of both TNF-α and IL-6 in G3 and G4 were still significantly higher (p<0.05) than the control group (G1) as seen in Figure 2.

**Figure 3.**
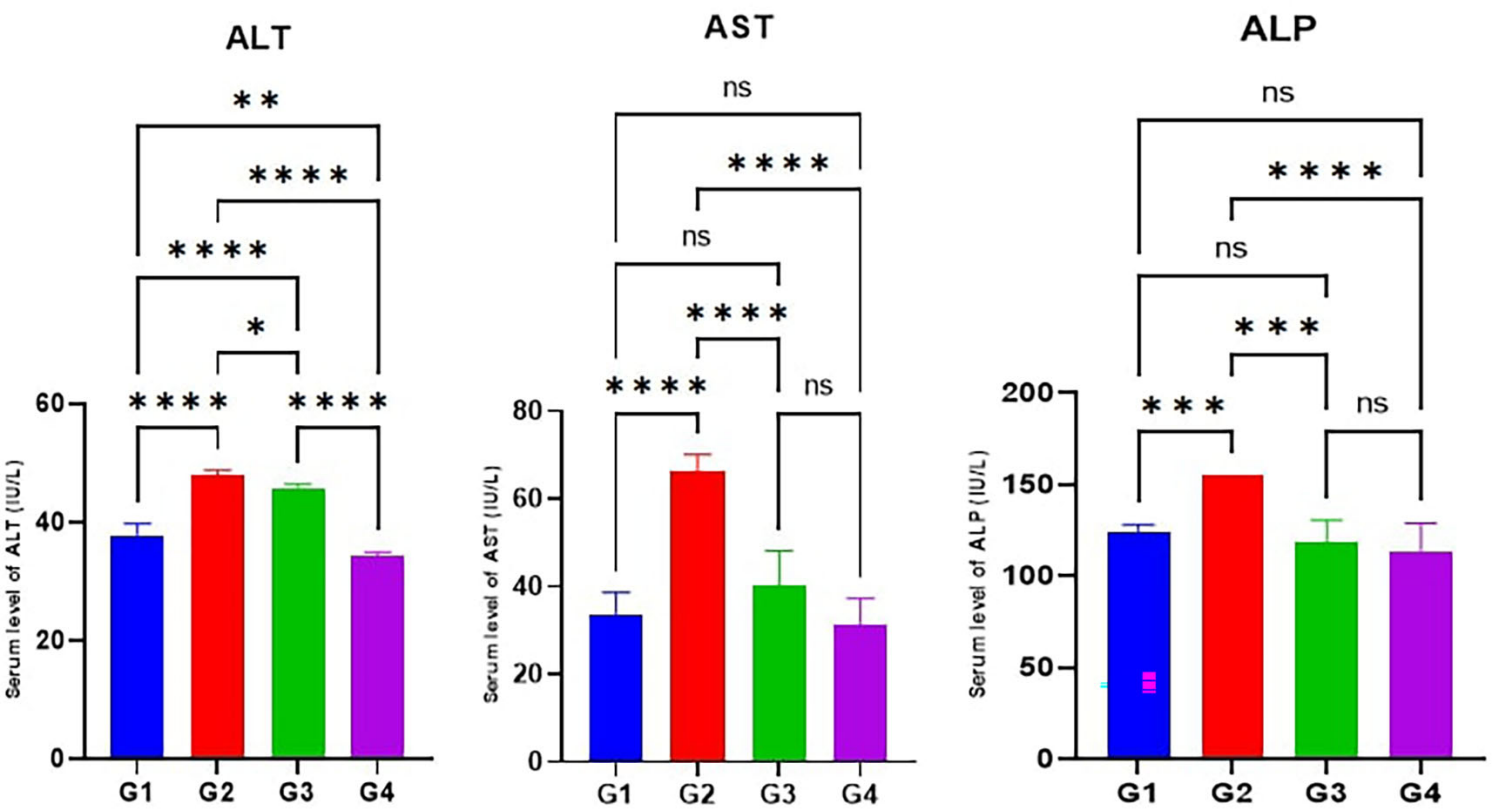
Effects of studied medications on hepatic enzymes AST, ALT, and ALP. Data are indicated as mean ±SD; ns (non-significant) = p > 0.05; **= significant differences (p < 0.01); ****= significant differences (p < 0.0001).

According to the results of the current study, the serum levels of AST, ALT, and ALP in the MTX induction group (G2) were significantly higher (p<0.05) than the control (G1). Furthermore, the serum levels of AST, ALT, and ALP in Juniperus Macrocarpa extract-treated groups G3 and G4 were significantly lower (p<0.05) than the MTX induction group (G2). Also, the serum levels of ALT in Juniperus Macrocarpa extract-treated groups G4 (200 mg/kg) were significantly lower (p<0.05) than in Macrocarpa extract-treated groups G3(100 mg/kg) While there was an insignificant difference (p>0.05) between G3 and G4 in the serum levels of AST and ALP. However, the serum levels of **ALT** in both G3 and G4 were still significantly higher (p<0.05) than in the control group (G1) as illustrated in Figure 3.

**Figure 4.**
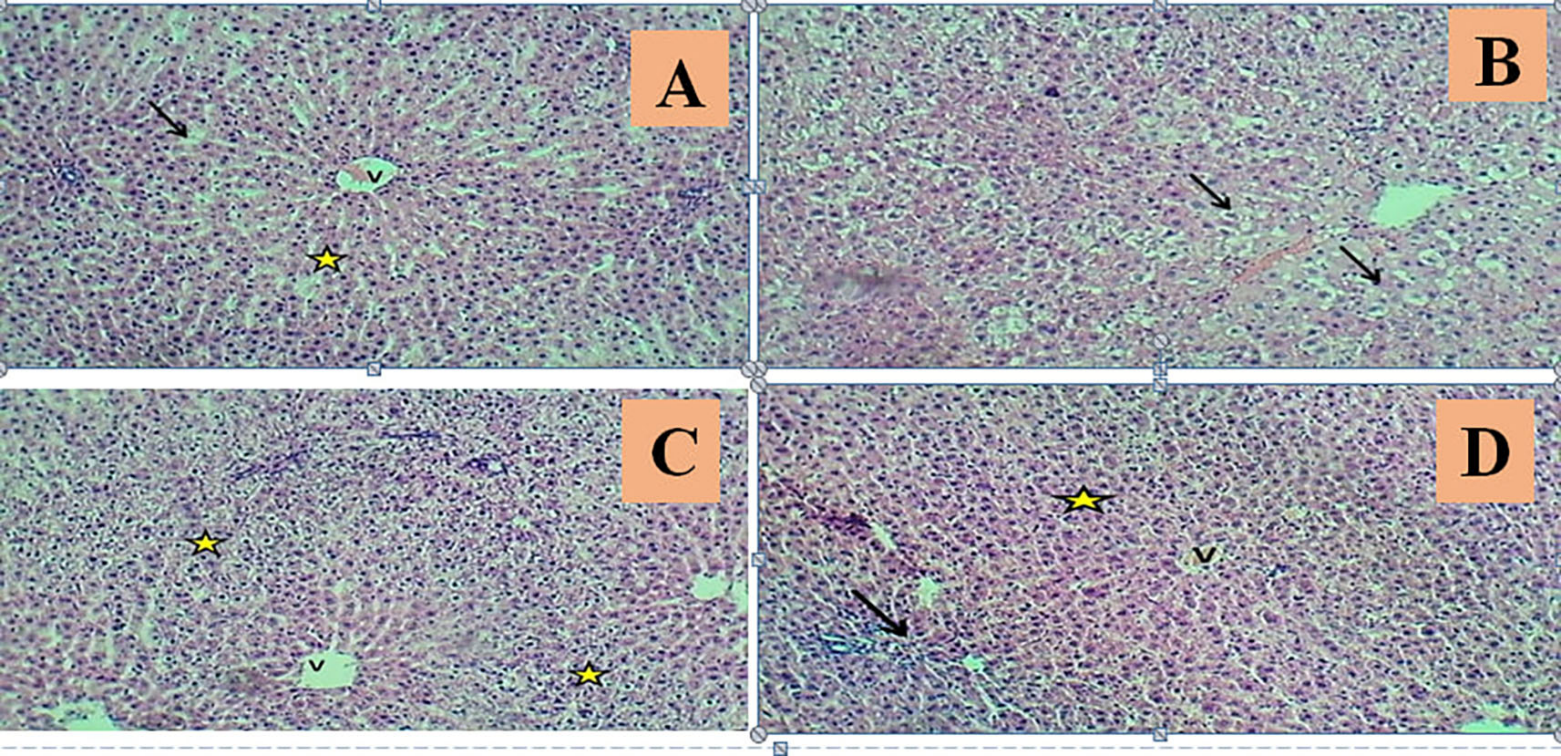
Effects of studied medications on hepatic histopathological changes. Histological photomicrographs obtained by processing rats’ livers from control (A), MTX induction (B), Juniperus Macrocarpa pretreatment (100 mg/kg) (C), and Juniperus Macrocarpa treatment (200 mg/kg) (D) groups. The control group shows the normal appearance of central vein (V), sinusoid (Black arrows) & normal arrangement of hepatic cords (Asterisk). The MTX induction group shows severe sinusoidal congestion with marked cellular swelling of hepatocytes with little necrosis (Arrows) and markedly disarrangement of hepatic cords. Regarding low-dose Juniperus Macrocarpa pretreatment, liver sections showed normal central vein (V), a normal arrangement of hepatic cords with marked zonal cellular swelling with little necrosis (Asterisks). Regarding high-dose Juniperus Macrocarpa pretreatment, liver sections showed mild congestion of the central vein (V) and a normal appearance of the portal triad (Arrow), with normally arranged hepatic cords (Asterisk), H&E stain.100×.

According to Figure 4, liver sections from the control group (G1) had normal central veins, hepatic cords, sinusoids, hepatocytes, and kupffer cells. In the MTX-induction group (G2), liver histopathology showed mild dilatation with central venous congestion, cellular and sinusoids, and localized necrosis with MNC aggregations. Furthermore, severe sinusoidal congestion with marked cellular swelling of hepatocytes and marked disarrangement of the hepatic cords coupled with fibroplasia and marked proliferation of cholangiocytes ( Figure 4). Pretreatment with low-dose Juniperus Macrocarpa extract 100 mg/kg/day (G3) preserved the normal liver structure as histopathological sections revealed a normal central vein with a normal arrangement of the hepatic cords. However, marked zonal cellular swelling with nuclear pyknosis and little necrosis are seen. Furthermore, pretreatment with Juniperus Macrocarpa extract 200 mg/kg/day (G4) resulted in sections with mild congestion of the central vein and hepatic sinusoids, a normal appearance of the hepatocytes arrangement that revealed a feature of bi-nucleated. The portal triad revealed normal cytoarchitecture and fibrous tissue. Other figures were similar to those of the control group.

## Discussion

MTX is the main treatment for various leukemia and lymphoma subtypes. ^88, 89^ Adverse responses limit the therapeutic use of this medication. ^90^ MTX was also utilized to control a range of inflammatory illnesses, notably psoriasis, vasculitis, and ulcerating colitis, as well as to terminate ectopic pregnancies on specific occasions. ^91– 97^ MTX’s most common and dangerous side effect is hepatotoxicity. ^98^ Previous research demonstrates that MTX produces several forms of harm in both humans and rats, including hepatitis, cirrhosis, elevated liver enzyme levels, gastrointestinal toxicity, and nephrotoxicity. ^99^ The primary center of metabolism, the liver performs a variety of tasks including producing biochemical enzymes, detoxifying the body, and producing vital proteins for blood clotting. ^100^

Numerous investigations have shown that MTX and its metabolites cause hepatocyte inflammation, oxidative stress, fibrosis, and apoptosis. ^35, 101^ The current study investigated whether Juniperus Macrocarpa extract may reduce MTX’s cytotoxic effect on liver cells. We measured blood antioxidant enzymes, inflammatory indicators, hepatic enzymes, and liver tissue histology to achieve our goal. We measured blood antioxidant enzymes, inflammatory indicators, hepatic enzymes, and liver tissue histology to achieve our goal.

According to the results of the current study, the level of antioxidant enzymes (SOD and GPx) in the serum is significantly reduced by MTX administration as compared to the control group. This finding agrees with previously published studies. ^102^ Nrf2 regulates antioxidant enzyme synthesis and is released from Kaap1, transcripted in the nucleus, and increases antioxidant enzyme synthesis during oxidative stress. ^102, 103^ Multiple studies demonstrated that Nrf2 mRNA levels are significantly downregulated in MTX-induced hepatotoxicity compared to the normal control group. ^104^ Treatment with 100 mg/kg and 200 mg/kg of Juniperus Macrocarpa extract as in groups G3 and G4 showed increased levels of antioxidant enzyme as compared to the MTX-induction group (G2). These effects of Juniperus Macrocarpa are attributed to its high polyphenols, flavonoid, and catechins compounds as indicated in the preliminary screening study. ^39^ These compounds are well known for their antioxidant potential which is described by several published articles. ^105^

According to the literature, the principal flavonoids present in Juniperus Macrocarpa extract were rutin, catechin, quercitrin, epicatechin, luteolin, and naringenin. ^39^ While polyphenolic compounds present were protocatechuic acid and gallic acid. ^39^ The principal mechanisms of flavonoid’s antioxidant activity are scavenging oxygen radicals, protecting against lipid peroxidation, and chelating metal ions. ^105^ Furthermore, it has been reported that these phytochemicals can induce Nrf _2_ expression or inhibit its proteasomal degradation by modifying the Nrf2–Keap1 complex which in turn increases antioxidant enzyme (SOD and GPx) synthesis. ^106, 107^

Additionally, G4 (200 mg/kg/day) exhibited statistically significantly greater SOD and GPx levels than G3 (100 mg/kg/day). The extract includes more pharmacologically active phytochemicals; therefore higher doses should be more helpful. Besides, in a study conducted to explore the protective effects of luteolin in MTX-induced hepatotoxicity, results showed that levels of SOD and GPx in the hepatic tissue were significantly increased coupled with upregulation in the Nrf2 gene expression. ^108^ Furthermore, to investigate the protective effects of gallic acid against MTX-induced hepatotoxicity, it was found that the tissue levels of antioxidant enzymes (SOD and GPx) were significantly elevated compared to untreated groups owing to their ROS scavenging ability. ^109, 110^

Results also demonstrated that MTX therapy increased pro-inflammatory cytokines in the blood. Another study found similar results. ^111^ MTX-induced inflammation is caused by increased ROS generation, which activates NF-κB in Kupffer cells. ^112^ NF-κB activation leads to the transcription of pro-inflammatory cytokines such as TNF-α and IL-6. Multiple hepatotoxic medications have been linked to elevated liver NF-κB expression and TNF- **α** levels. ^113, 114^ While the Juniperus Macrocarpa treatment groups G3 and G4 showed reduced levels of inflammatory cytokines as compared to the MTX-induction group (G2); these findings are attributed to the phytochemical composition of Juniperus Macrocarpa as it is abundant in polyphenols and flavonoid compounds which have antioxidant and anti-inflammatory effects. ^39^ The antioxidant properties of Juniperus Macrocarpa extract mitigates the oxidative stress induced by MTX leading to a reduction in the activation of oxidative stress response of NF-κB and reduced the expression of pro-inflammatory cytokines. ^115^ Additionally, it was found that G4 had lower **TNF-α and IL-6** levels compared to G3 which is statistically significant. As the extract contains more pharmacologically active phytochemicals, bigger dosages should have more therapeutic potential.

As mentioned previously, MTX-induced hepatic damage is associated with cellular death and necrosis coupled with the release of hepatic enzymes into the circulation. ^116^ This resulted in a significant elevation of the AST, ALT, and ALP serum levels in MTX-induction (G2) as compared to the normal control group. However, Juniperus Macrocarpa pretreatment groups G3 and G4 had lower AST, ALT, and ALP levels than the MTX-induction group (G2). This indicates less damage to the hepatocyte and the hepatoprotective effects of Juniperus Macrocarpa extract. An explanation for this finding could be made based on the previous result represented by the ability of the extract to enhance the level of antioxidant enzymes and counteract the state of oxidative stress. This leads to a decrease in lipid peroxidation and the proposed damage to the cell membrane. Controlling oxidative stress diminishes NF-κB pathway activation, TNF-α, and IL-6 production, leading to mitigated inflammatory response. These actions are attributed to the phytochemical composition of the Juniperus Macrocarpa extract represented by polyphenols and flavonoid content leading collectively to less damage to the hepatocytes and reduced serum levels of AST, ALT, and ALP. ^39^ Furthermore, G4 had lower AST, ALT, and ALP levels compared to G3 which is only significant in the case of ALT. This is expected as higher doses of the extract have more concentration of pharmacologically active phytochemicals leading to enhanced therapeutic potential. Regarding the hepatic enzymes, ALT is solely produced by the liver so it is more reliable to assess the magnitude of liver damage. ^117^ In research on apigenin protective effects against MTX-induced hepatotoxicity, serum AST, ALT, and ALP had dramatically decreased upon pretreatment. These findings were attributed to the antioxidant effects of apigenin and its protection against lipid peroxidation which preserves cell membrane and hepatic cells from necrosis. ^118^ Similarly, epicatechin pretreatment significantly reduces serum levels of AST, ALT, and ALP owing to their anti-inflammatory and antioxidant properties which protect hepatocytes from damage. ^119^

The histopathological damage score for G2 was significantly higher (p<0.05) than the control group (G1). Hepatic sections stained with H and E revealed that the MTX induction group (G2) suffered from several histopathological alterations including dilatation and congestion of the hepatic sinusoids, inflammation, hepatocyte degeneration, vacuolization, and fibrosis. The observed changes agree with previously obtained biochemical results represented by a reduction in the levels of antioxidant enzymes, elevation of the circulatory levels of hepatic enzymes (AST, ALT, and ALP), and proinflammatory cytokinins. These results agree with previous studies which declared that MTX causes focal necrosis, dilatation in the central vein, accompanied by inflammatory cells, many bodies of apoptosis with dense cytoplasm, and peripheralized pyknotic nuclei. ^120, 121^ Furthermore, MTX-induction (G2) showed a high degree of cholangiocyte infiltration indicating damage to the bile duct which is compatible with elevated levels of ALP previously obtained. This effect was also obtained in another research. ^122^ This finding is explained by damage to the hepatic cells caused by MTX resulting in oxidative stress and ROS coupled with MTX-induced downregulation in the Nrf2 gene expression, eventually leading to a reduction in the activities of the antioxidant enzymes. ^35, 102^ ROS causes damage to the cell membrane and induces an inflammatory response by activation of NF- *κ*B and increased expression of proinflammatory cytokines (TNF-α and IL-6). ^123– 125^

In apoptosis, MTX increased Bax and decreased Bcl-2. ^126^ In MTX hepatotoxicity, oxidative stress causes Bax translocation to the outer mitochondrial membrane, which increases mitochondrial permeability and cytochrome c release into the cytosol, activating downstream effector caspases. ^127^ HSC activation is also caused by oxidative stress and ROS. ^128^ HSC activation promotes substantial portal collagen and fibrosis deposition. ^129^ To test whether pretreatment with Juniperus Macrocarpa extract (100 and 200 mg/kg) lowers MTX toxicity, we gave rats MTX after treatment. The G3 and G4 groups had considerably reduced histopathological damage scores (p<0.05) than the MTX-induction group (G2). The fact that the Juniperus Macrocarpa extract-pretreated groups experienced less MTX-induced liver damage suggests their ability to maintain a respectable level of hepatic integrity. Additionally, there was less inflammatory cell infiltration and fibrosis than the MTX-treated group with the median hepatic damage score for both G3 and G4 significantly less than the MTX-induction group G2. The histopathological data presented in this section are consistent with earlier studies that showed elevated activity of antioxidant enzymes and a decrease in serum levels of liver enzymes. However, it was insignificantly different between G3 and G4 where the animals were given low and high doses of Juniperus Macrocarpa extract despite the lower mean value of the hepatic damage score in G3 which reflects low sample size. The found hepatoprotective activity may be attributed to their antioxidant potential against reactive oxygen and nitrogen species, which inhibit lipid peroxidation and liver cell necrosis or apoptosis. The phenol-rich ethyl acetate fraction of the ethanolic extract of J. communis leaves showed a strong hepatoprotective effect against paracetamol-induced hepatotoxicity in rats without cytotoxicity, according to prior research. ^130^ The Limitations of the current study could be summarized by a small number of animals used and the lack of estimation of the exact molecular mechanism through which Juniperus Macrocarpa extract exerts its hepatoprotective effect. Estimation of Malondialdehyde (MDA) tissue levels, being the product of lipid peroxidation and the expression level of nuclear factor erythroid 2-related factor 2 (Nrf2) mRNA is usually used to predict the pathways of enhancing antioxidant activity while the nuclear factor kappa (NF-kB) mRNA to estimate the anti-inflammatory activity of the extract. Additionally, the expression levels of Bax and caspase-3 as a pro-apoptotic protein and Bcl-2 as an anti-apoptotic mRNA are recommended for further investigation to estimate the exact mechanism by which Juniperus Macrocarpa extract exerts its antiapoptotic effects.

## Conclusions

The present study showed that pretreatment with Juniperus Macrocarpa extract can attenuate severe alterations in hepatic biochemical markers and disruptions of its histological structure.

## Ethics statement

The ethics council at the College of Medicine/University of Baghdad gave permission for this study. The obligations and standards outlined in the Declaration of Helsinki were rigorously followed when developing this research. On December 3, 2023, a local ethical authority verified the necessary papers and client information to validate the experiment’s techniques (document authorization number UoB.Med.36). All efforts were made to ameliorate the total number of animals used in the testing and their suffering by housing them in separate sterile containers with an expanded wire-meshed ground underneath suitable locations, ensuring a period of twelve hours of light and darkness, and administering anesthetic drugs to alleviate any encountered discomfort or pain.

## Author contributions

**Shahad Hassan Hadi** prepared the final copy of the paper, which encouraged participation in the project’s approach, gave funding, monitored the examination, and funded supplies. **Mohammed Qasim Yahya Malallah A. Al-atrakji** accomplished the roles of invention, validation, and supervision and provided the executable language of the updated paperwork as well as statistical data calculation and electronic reinforcement.

## Data Availability

Figshare: Ameliorative impact of Juniperus Macrocarpa extract on methotrexate-induced hepatic damage in rats, https://doi.org/10.6084/m9.figshare.27629925.v2. ^131^ Data are available under the terms of the Creative Commons Attribution 4.0 International license (CC-BY 4.0). The article adheres to the ARRIVE guidelines. Figshare: Ameliorative impact of Juniperus Macrocarpa extract on methotrexate -induced hepatic damage in rats ( 10.6084/m9.figshare.27629925). ^132^ Data are available under the terms of the Creative Commons Attribution 4.0 International license (CC-BY 4.0).
