# Supplementary material for: The role of Juniperus Macrocarpa extract as anti-inflammatory and antioxidant on methotrexate-induced acute liver injury in rat model
Source: F1000Res. 2025 Jan 27;14:131. [Version 1] doi: 10.12688/f1000research.158839.1 (PMC11894369; doi:10.12688/f1000research.158839.1)
Supplement: Peer review history

# Reviewer report: Reviewer response for version 1

**DOI:** 10.5256/f1000research.174485.r368333

**Published:** 2025-03-10

**Authors:** Yaarub Sadiq

**Affiliations:** 1 Pharmacology and pharmaceutical sciences, University of Sharjah, Sharjah, Sharjah, United Arab Emirates

Congratulations on the outstanding effort accomplished by the author team. My main concern I want to highlight is that the authors mentioned in the abstract that Group 2 (MTX) got an intraperitoneal single dose of MTX (20 mg/kg) for two weeks. While in the experimental design, they mentioned that Group II (Induction) group(n=5): rats were administrated pre-treatment with 1 mL of distilled water orally for 13 days by oral gavage and 20mg/kg of Methotrexate intraperitoneally on day 13 of the study. However, there is inconsistency in the dose of methotrexate; is it continued for two weeks, or is it given as a single dose at the end of the study?

 In addition, the selection of suitable abbreviations in the abstract and text should be consistent throughout; for example, (MTX vs. Methotrexate). In the same vein, the scientific nomenclature of the natural herb species should be edited to its right designation. The genus name is consistently capitalized and written first. The names of species are consistently *italicized* in lowercase: GENUS *species*. Also, in the discussion part, the authors abbreviated the genus name of the same herb with a different species (J. communis). The labeling should be standardized (J. vs. Juniperus).

Is the work clearly and accurately presented and does it cite the current literature?

Yes

If applicable, is the statistical analysis and its interpretation appropriate?

Yes

Are all the source data underlying the results available to ensure full reproducibility?

Yes

Is the study design appropriate and is the work technically sound?

Yes

Are the conclusions drawn adequately supported by the results?

Yes

Are sufficient details of methods and analysis provided to allow replication by others?

Yes

Reviewer Expertise:

Brain damage, hepatorenal damage, gastrointestinal diseases, myocardial ischemia/reperfusion, eye diseases and health.

I confirm that I have read this submission and believe that I have an appropriate level of expertise to confirm that it is of an acceptable scientific standard.

# Reviewer report: Reviewer response for version 1

**DOI:** 10.5256/f1000research.174485.r368325

**Published:** 2025-03-08

**Authors:** Hayder Ridha-Salman

**Affiliations:** 1 Al-Mustaqbal University, College of Pharmacy, Babylon, Hilla, 51001, Iraq

1.The authors could enhance their introduction by incorporating more details pertaining to the rationale behind the selection of Juniperus macrocarpa extract as an alternative to other herbal remedies.

 2. Please provide details regarding pro-inflammatory and oxidative stress biomarkers. This could potentially support the importance of the biochemical indicators utilized in your research and assist readers in understanding the protective rationale behind Juniperus macrocarpa's anti-inflammatory and antioxidant activities.

 3. In the methodology section, please include a new heading for the evaluation of oxidative and inflammatory biomarkers.

 4. Please incorporate the value of n=? into figure captions.

 5. It would be advantageous to incorporate more details regarding the preparation of Juniperus macrocarpa extract.

 6. The statistical software version deserves to be described for better reproducibility.

Is the work clearly and accurately presented and does it cite the current literature?

Yes

If applicable, is the statistical analysis and its interpretation appropriate?

Yes

Are all the source data underlying the results available to ensure full reproducibility?

Yes

Is the study design appropriate and is the work technically sound?

Yes

Are the conclusions drawn adequately supported by the results?

Yes

Are sufficient details of methods and analysis provided to allow replication by others?

Yes

Reviewer Expertise:

Pharmacology, Toxicology, Antioxidant agents, Anti-inflammatory drugs, immunomodulators, Natural products, dermatological therapies, skin diseases and treatments.

I confirm that I have read this submission and believe that I have an appropriate level of expertise to confirm that it is of an acceptable scientific standard, however I have significant reservations, as outlined above.

# Reviewer report: Reviewer response for version 1

**DOI:** 10.5256/f1000research.174485.r368331

**Published:** 2025-03-04

**Authors:** Ahemed Abu-Raghif

**Affiliations:** 1 Al-Nahrain University, Baghdad, Iraq; 2 Pharmacology, Al-Nahrain University, College of Medicine, Baghdad, Baghdad, Iraq

• The species name of the medicinal plant is not written correctly and must adhere to proper scientific nomenclature as follows: Juniperus macrocarpa.

 • The Materials and Methods section should start with a brief description of the study, including the type, date, and location of the research.

 • The phrase "Assessment of hepatic marker enzymes" should be replaced with "Assessment of hepatic enzymes," as hepatic markers is a general term that may cover biomarkers other than liver enzymes.

 • In the discussion section, it is recommended to include a suggestion regarding the isolation of the most effective part of the plant, which can be used in future liver protection research.

Is the work clearly and accurately presented and does it cite the current literature?

Yes

If applicable, is the statistical analysis and its interpretation appropriate?

Yes

Are all the source data underlying the results available to ensure full reproducibility?

Yes

Is the study design appropriate and is the work technically sound?

Yes

Are the conclusions drawn adequately supported by the results?

Yes

Are sufficient details of methods and analysis provided to allow replication by others?

Yes

Reviewer Expertise:

Pharmacology and Toxicology

I confirm that I have read this submission and believe that I have an appropriate level of expertise to confirm that it is of an acceptable scientific standard, however I have significant reservations, as outlined above.
